# Treatment of malignant pleural effusions: the case for localized immunotherapy

**DOI:** 10.1186/s40425-019-0590-4

**Published:** 2019-04-18

**Authors:** Albert D. Donnenberg, James D. Luketich, Rajeev Dhupar, Vera S. Donnenberg

**Affiliations:** 1 Department of Medicine, Division of Hematology-Oncology, University of Pittsburgh School of Medicine, Pittsburgh, PA USA; 2 Department of Cardiothoracic Surgery, Division of Thoracic and Foregut Surgery, University of Pittsburgh School of Medicine, Pittsburgh, PA USA; 3 Department of Pharmaceutical Sciences, University of Pittsburgh School of Pharmacy, Pittsburgh, PA USA; 4 Departments of Medicine and Infectious Disease and Microbiology, University of Pittsburgh, School of Medicine and Graduate School of Public Health, Hillman Cancer Center, Research Pavilion, 5117 Centre Ave, Pittsburgh, PA 15213 USA; 5 Departments of Cardiothoracic Surgery and Pharmaceutical Sciences, University of Pittsburgh, Schools of Medicine and Pharmacy, Hillman Cancer Center, Research Pavilion, 5117 Centre Ave, Suite 2.42, Pittsburgh, PA 15213 USA

**Keywords:** Malignant pleural effusion, Immunotherapy, Pleural infiltrating T cells (PIT), Tumor associated macrophages (TAM)

## Abstract

Malignant pleural effusions (MPE) are a common terminal pathway for many cancers, with an estimated United States incidence of more than 150,000 cases per year. MPE is an aggressive disease with a uniformly fatal prognosis and a life expectancy of only 3 to 12 months. The development of an effective targeted therapy represents a pressing unmet need. This commentary focuses on how cellular and humoral components condition the pleural space as a tumor-promoting, wound-healing environment. Despite an abundance of potential antigen presenting and effector cells in the pleura, their physical isolation by the mesothelial barrier, the concentration of cytokines and chemokines driving the epithelial to mesenchymal transition (EMT) and M2 /Th-2 polarization, suppress tumor-specific immune effector responses. We argue that local immune repolarization must precede either immune checkpoint or cellular therapy to successfully eradicate pleural tumor. We further hypothesize that, because of its cellular content, a repolarized pleural space will provide an effective immune environment for generation of systemic anti-tumor response.

## Introduction

At advanced stages, metastatic cancer infiltrates thoracic lymph nodes as well as the lining of the chest cavity, known as the pleura. When this occurs, the normal cycle of fluid secretion and absorption is interrupted, resulting in fluid collection and compression of the lung. The fluid, which is composed of serous proteins, cancer cells, and lymphoid and myeloid immune cells, is termed malignant pleural effusion (MPE). Accumulation of fluid results in symptoms that range from cough to life threatening dyspnea and hypoxia, but it is the aggressive nature of the tumor to which the patient ultimately succumbs. Epithelial cancers account for about 80% of patients receiving interventions and life expectancy ranges from 3 to 12 months [1] . Despite the fact that the incidence of MPE in the United States exceeds 150,000 cases per year [2], and despite the fact that a wide range of systemic and localized therapeutic approaches have been tested, current best practice is limited to palliation by drainage [3]. It is our contention that the interactions that occur between tumor and immune cells in the confines of the pleural space make it an incubator promoting an epithelial to mesenchymal transition (EMT) and the emergence of the most aggressive drug-resistant neoplastic cells. This commentary makes the case for an intrapleural immunotherapeutic approach for patients with MPE. Our growing appreciation of the immune environment of the pleural space and of the complex interplay between tumor and immune cells suggests a more rational immunotherapeutic approach to treat this condition.

### Localized immunotherapy

The concept of immune system activation in the setting of thoracic malignancy dates to the 1970’s, when investigators noted improved survival in patients with empyema after resections for lung cancer [4], justifying largely unsuccessful trials of intrapleural Baccilus Calmette-Guérin (BCG) [5] and other bacterial antigens. Direct instillation of the recombinant cytokines interferon ɣ [6], interferon α2b [7] and IL-2 [8, 9] have also been tested. Intrapleural IL-2 was well tolerated in non-small cell lung cancer (NSCLC). Further, intrapleurally administered IL-2 levels were 6000-fold higher than in the plasma [8], indicating that locally administered IL-2 (formula weight = 15.5 kDa) is sequestered in the pleural space. This is a very important observation, since other large molecule biologicals, such as antibodies can be expected to be similarly concentrated when administered directly to the pleura. Although intrapleural IL-2 cleared effusions in 28 of 31 patients studied [9], and partial responses were seen with other cytokine modalities, the median time to progression ranged from days [7] to months [8]. Thus instillation of high-dose Th1 associated cytokines was insufficient by themselves to overcome the immunosuppressive environment of the pleural space.

### Therapeutic use of pleural-infiltrating T cells (PIT)

A recent clinical trial described the use of tumor infiltrating lymphocytes (TIL) derived from MPE and malignant ascites in combination with cisplatin [10]. Effusion-derived TIL provided longer progression-free survival and better quality of life than cisplatin alone. The combination of immunotherapy and chemotherapy is somewhat counterintuitive since many cytotoxic agents inhibit cell proliferation, an important aspect of adaptive immunity. However, regulatory T cells (Treg) have been shown to be more susceptible to platinum-based combination chemotherapy than conventional CD4+ T cells [11], potentially providing a release from Treg-mediated suppression of anti-tumor immunity. Effusion-derived TIL have several important advantages over TIL derived from primary tumors or biopsies of solid metastases: [1] The T-cell yield from MPE or ascites is orders of magnitude higher than from biopsies, so the number of passages required is lower, and culture time is shorter; [2] Malignant effusion pleural T cells (PIT) represent a cross-section of all TIL, whereas TIL derived from solid tumors display spatial heterogeneity and can differ with respect to function and specificity depending on the biopsy location [12]. Owing to their abundance, it may be possible to prime PIT by short term ex vivo exposure to activation signals, and reinstill them without expansion. The resulting cells would not be cytokine addicted as are conventional TIL and would not require administration of high dose IL-2 for their survival.

### Immune checkpoint inhibitors in MPE

PD-L1 is expressed on malignant mesothelioma [13] and other malignancies and is therefore potentially targetable by anti-PD-L1 antibodies. T cells from NSCLC MPE display increased expression of PD-1, TIM-3, and CTLA-4, when compared with non-malignant controls [14], possibly due to high levels of TGF-β in the effusion, secreted by PD-L1+ tumor-associated M2-macrophages.

### Toward efficacious localized immunotherapy therapy

It is becoming increasingly clear that both conventional and immunotherapeutic attempts have failed in MPE because the pleural space is a sequestered environment in which tumor cells and immune cells interact to the benefit of the tumor. In the pleural space, wound-healing cytokines and chemokines are concentrated, and juxtacrine interactions of tumor, macrophages and mesothelial cells are favored by their proximity. The result is the perpetuation of a wound-healing environment in which T-cell effectors are suppressed or killed, and macrophages are channeled to an M2 program that assists angiogenesis and metastasis, all culminating in the promotion of an aggressive and invasive EMT tumor phenotype.

### Sequestered environment

The pleural space represents a sequestered local environment formed by mesothelial cells joined by tight junctions [15]. Protein biologics such as IL-2 remain highly concentrated when administered intrapleurally, with local concentrations thousands of times higher than that of the plasma [8]. Protein movement from the plasma to the pleura is also impeded, albeit to a lesser extent, and the pleural effusion to plasma ratio of protein concentrations is inversely related to their molecular weight [16]. This is highly relevant to systemic administration of antibody therapeutics, which predictably would not pass easily into the pleural space, abdominal cavity, or interstitial spaces [17].

### The pleural secretome

The cell-free serous component of MPE contains an array of cytokines and chemokines [18]. The majority of the secreted cytokines in MPE are Th2-like and include IL-10 [19], VEGF [20] and TGFβ [21], further promoting the wound-healing milieu to the detriment of an anti-tumor effector response. Interestingly, the pleiotropic cytokine IL-6 and its soluble receptor component sIL-6Rα are among the most abundant cytokines in MPE [22]. IL-6 is produced by the tumor [23] and also by pleural mesothelial cells [24] and stromal cells [25]. IL-6 plus IL-10 upregulate PD-L1 expression in tumor cells [26]. IL-6 signal transduction is mediated through a receptor complex consisting of IL-6Rα (CD126) and IL-6Rβ (CD130). IL-6Rβ is expressed ubiquitously, but IL-6Rα expression is restricted chiefly to leukocytes and hepatocytes. In normal physiology, IL-6 mediates powerful systemic effects by trans-signaling, which occurs when IL-6 binds to soluble IL-6Rα and complexes with membrane-bound IL-6Rβ [27]. IL-6 trans-signaling has been shown to promote aggressive tumor behavior and progression in malignant ascites of ovarian cancer [28] and in breast cancer pleural effusions [23], and promotes EMT in non-small cell lung cancer [29], making it an attractive therapeutic target. Tocilizumab, a monoclonal antibody directed against IL-6Rα, is licensed for the treatment of rheumatoid arthritis, and has been used experimentally to treat cancer-associated cachexia [30] and cytokine release syndrome [31]. Intrapleural administration may have profound effects on the polarization of the pleural immune environment with minimal systemic effects.

### Juxtacrine interactions

The proximity and high concentration of T cells, macrophages, mesothelial cells and tumor in the pleural space favors cell-cell contact and juxtacrine signaling. Examples include the promotion of EMT by binding of CD90 and EphA4 on the tumor to CD11b and Ephrin on macrophages, respectively [23]. Similarly, PD-L1 and PD-L2 expressed on tumor and pleural macrophages bind to PD-1 on T cells, promoting anergy, development of induced regulatory T cells (iTregs) and apoptosis [32]. Other ligands expressed on pleural tumor, such as CEACAM1 which binds to TIM-3, may interact with immune checkpoint receptors expressed on PIT. MPE, which are routinely therapeutically drained, provide a unique window into interactions that are more difficult to observe in other metastatic settings.

## Conclusion

**Fig. 1:**
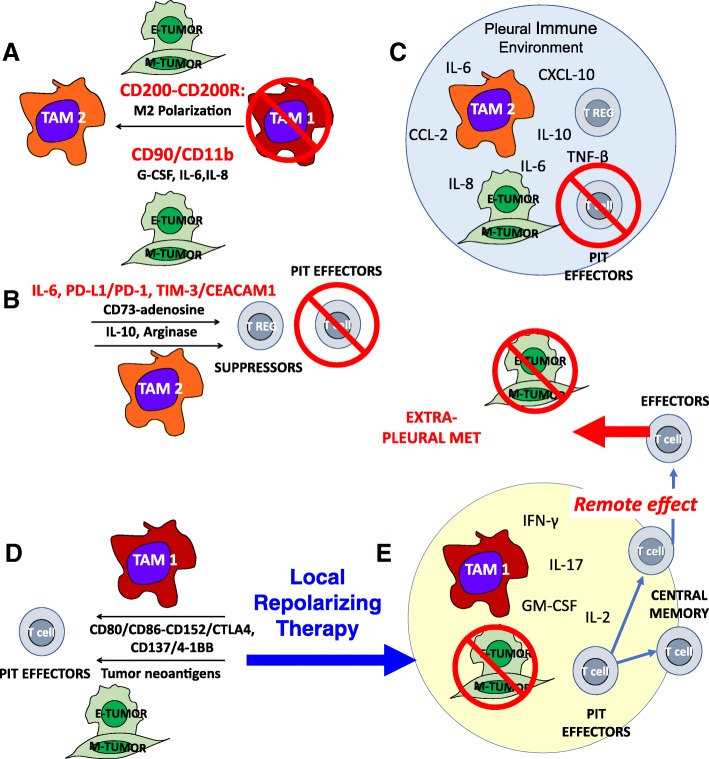
The interactions of tumor, TAM, and T-cells are highly dependent on the local immune environment. Tumors can evade an immune response, even when all of the required components are abundant and in close proximity. Panel **a**: Tumor effects on TAM. Tumor, particularly tumor that has undergone EMT, amplifies and maintains TAM M2-like wound healing polarization. TAM are recruited with tumor-secreted G-CSF and polarized through CD200/CD200R and CD90/CD11b interactions and tumor secreted IL-6 and IL-8. Panels **b** and **c**: Tumor and TAM polarization effects on T cells. M2 TAM polarization favors T-cell suppression through cytokines and programmed death ligand-induced apoptosis (**b**). The pleural space is isolated from the systemic circulation, permitting the maintenance of very high local cytokine and chemokine levels. The encircled space (Panel **c**) represents the isolated pleural immune environment in which potential effector cells are potently suppressed. It should be possible to repolarize the pleural immune environment with local delivery of cytokines, activation signals, antibody-based therapeutics and ex vivo activated PIT, tipping the balance in favor of the immune system. Panel **d**: Local repolarizing therapy results in M1 TAM polarization, providing presentation of tumor neo-antigens and costimulatory signals, culminating in the generation of tumor-specific effector T cells. Panel **e**: Once the immune blockade has been breached, antigen presenting cells and T cells can traffic to the draining lymph nodes where responding T cells can proliferate and differentiate. Once this has occurred, tumor specific T cells can be expected to home to other sites of metastasis

Despite continuing efforts to provide effective systemic and localized cytotoxic and immune-based therapies, there is currently no effective treatment for malignant pleural effusions. We argue that the pleural space, because of the physical barrier provided by the mesothelium, acts like a bioreactor in which carcinoma cells, TAM, PIT and stroma interact (Fig. 1). The concentration of wound-healing cytokines and chemokines and environmental polarization resulting from these multi-way feedback interactions promote EMT and aggressive tumor behavior, and thwarts anti-tumor immune effector responses by multiple distinct and probably synergistic mechanisms. The benefit of intrapleural administration is that high molecular weight biologicals are sequestered in the pleural space [8, 33] by the same mechanism that allows the accumulation of high concentrations of locally secreted cytokines. Thus, repolarizing treatment combinations that would have unacceptable dose-limiting toxicities when given systemically, may have more acceptable toxicity profiles when administered directly to the pleural space at a fraction of the systemic dose.

The remarkable progress of cancer immunotherapeutics over the last decade demands that we devise new methods to *treat* rather than palliate malignant pleural effusions. Recognizing the pleural space as a sequestered environment in which all the components required for an effective anti-tumor response are present, but conscripted into a wound-healing mode, we argue that a combination of local repolarizing therapy in combination with immune checkpoint blockade and therapeutic effector cells may be sufficient to turn a dire clinical situation to therapeutic advantage.

## Abbreviations

CEACAM: Carcinoembryonic antigen-related cell adhesion molecule
CTLA-4: Cytotoxic T-lymphocyte-associated protein 4
EMT: Epithelial to mesenchymal transition
FcR: Fraction crystalizable receptor
IL: Interleukin
iTreg: induced regulatory T cell
M2: M2-type macrophage polarization
MPE: Malignant pleural effusion
PD: Programmed death
PD-L: Programmed death ligand
PIT: Pleural infiltrating T cell
TAM: Tumor associated macrophage
TGF: Transforming growth factor
Th2: Th2-type T-cell polarization
TIL: Tumor infiltrating lymphocyte
TIM-3: T-cell immunoglobulin and mucin-domain containing-3
VEGF: Vascular endothelial growth factor

## References

[1] Clive AO, Kahan BC, Hooper CE, Bhatnagar R, Morley AJ, Zahan-Evans N, et al. Predicting survival in malignant pleural effusion: development and validation of the LENT prognostic score. Thorax. 2014. 10.1136/thoraxjnl-2014-205285 PMC4251306 25100651

[2] American Thoracic S (2000). Management of malignant pleural effusions. Am J Respir Crit Care Med.

[3] Warren WH, Kalimi R, Khodadadian LM, Kim AW (2008). Management of malignant pleural effusions using the Pleur(x) catheter. Ann Thorac Surg.

[4] Ruckdeschel JC, Codish SD, Stranahan A, McKneally MF (1972). Postoperative empyema improves survival in lung cancer. Documentation and analysis of a natural experiment. N Engl J Med.

[5] Bakker W, Nijhuis-Heddes JM, van der Velde EA (1986). Post-operative intrapleural BCG in lung cancer: a 5-year follow-up report. Cancer Immunol Immunother.

[6] Yanagawa H, Haku T, Hiramatsu K, Nokihara H, Takeuchi E, Yano S (1997). Intrapleural instillation of interferon gamma in patients with malignant pleurisy due to lung cancer. Cancer Immunol Immunother.

[7] Sartori S, Tassinari D, Ceccotti P, Tombesi P, Nielsen I, Trevisani L (2004). Prospective randomized trial of intrapleural bleomycin versus interferon alfa-2b via ultrasound-guided small-bore chest tube in the palliative treatment of malignant pleural effusions. J Clin Oncol.

[8] Goey SH, Eggermont AM, Punt CJ, Slingerland R, Gratama JW, Oosterom R (1995). Intrapleural administration of interleukin 2 in pleural mesothelioma: a phase I-II study. Br J Cancer.

[9] Castagneto B, Zai S, Mutti L, Lazzaro A, Ridolfi R, Piccolini E (2001). Palliative and therapeutic activity of IL-2 immunotherapy in unresectable malignant pleural mesothelioma with pleural effusion: results of a phase II study on 31 consecutive patients. Lung Cancer.

[10] Chu H, Du F, Gong Z, Lian P, Wamg Z, Li P (2017). Better clinical efficiency of TILs for malignant pleural effusion and ascites than cisplatin through Intrapleural and intraperitoneal infusion. Anticancer Res.

[11] Maeda K, Hazama S, Tokuno K, Kan S, Maeda Y, Watanabe Y (2011). Impact of chemotherapy for colorectal Cancer on regulatory T-cells and tumor immunity. Anticancer Res.

[12] Mani NL, Schalper KA, Hatzis C, Saglam O, Tavassoli F, Butler M (2016). Quantitative assessment of the spatial heterogeneity of tumor-infiltrating lymphocytes in breast cancer. Breast Cancer Res.

[13] Khanna S, Thomas A, Abate-Daga D, Zhang J, Morrow B, Steinberg SM, et al. Malignant mesothelioma effusions are infiltrated by CD3+ T cells highly expressing PD-L1 and the PD-L1+ tumor cells within these effusions are susceptible to ADCC by the anti-PD-L1 antibody Avelumab. Journal of thoracic oncology : official publication of the International Association for the Study of Lung Cancer. 2016. 10.1016/j.jtho.2016.07.033 PMC5075512 27544053

[14] Li L, Yang L, Wang L, Wang F, Zhang Z, Li J (2016). Impaired T cell function in malignant pleural effusion is caused by TGF-beta derived predominantly from macrophages. Int J Cancer.

[15] Amasheh S, Markov AG, Volgin GN, Voronkova MA, Yablonsky PK, Fromm M (2011). Barrier function of human pleura mesothelium is constituted by tight junctions. FASEB J.

[16] Telvi L, Jaubert F, Eyquem A, Andreux JP, Labrousse F, Chretien J (1979). Study of immunoglobulins in pleura and pleural effusions. Thorax..

[17] Khaowroongrueng V, Jadhav SB, Fueth M, Otteneder MB, Richter W, Derendorf H (2017). Application of large-pore microdialysis in interstitial sampling of therapeutic monoclonal antibody. Clinical pharmacology in drug development.

[18] Psallidas I, Kalomenidis I, Porcel JM, Robinson BW, Stathopoulos GT (2016). Malignant pleural effusion: from bench to bedside. Eur Respir Rev.

[19] Chen Y-M, Yang W-K, Whang-Peng J, Kuo BI-T, Perng R-P (1996). Elevation of Interleukin-10 levels in malignant pleural effusion. Chest..

[20] Hooper CE, Elvers KT, Welsh GI, Millar AB, Maskell NA (2012). VEGF and sVEGFR-1 in malignant pleural effusions: association with survival and pleurodesis outcomes. Lung Cancer.

[21] Thomas R, Cheah HM, Creaney J, Turlach BA, Lee YCG (2016). Longitudinal measurement of pleural fluid biochemistry and cytokines in malignant pleural effusions. Chest..

[22] Dore P, Lelievre E, Morel F, Brizard A, Fourcin M, Clement C (1997). IL-6 and soluble IL-6 receptors (sIL-6R and sgp130) in human pleural effusions: massive IL-6 production independently of underlying diseases. Clin Exp Immunol.

[23] Lu H, Clauser KR, Tam WL, Frose J, Ye X, Eaton EN (2014). A breast cancer stem cell niche supported by juxtacrine signalling from monocytes and macrophages. Nat Cell Biol.

[24] Fujino S, Yokoyama A, Kohno N, Hiwada K (1996). Interleukin 6 is an autocrine growth factor for normal human pleural mesothelial cells. Am J Respir Cell Mol Biol.

[25] Malhotra D, Fletcher AL, Astarita J, Lukacs-Kornek V, Tayalia P, Gonzalez SF (2012). Transcriptional profiling of stroma from inflamed and resting lymph nodes defines immunological hallmarks. Nat Immunol.

[26] Mittendorf EA, Philips AV, Meric-Bernstam F, Qiao N, Wu Y, Harrington SM, et al. PD-L1 expression in triple negative breast Cancer. Cancer Immunol Res. 2014. 10.1158/2326-6066.CIR-13-0127 PMC4000553 24764583

[27] Scheller J, Chalaris A, Schmidt-Arras D, Rose-John S (2011). The pro- and anti-inflammatory properties of the cytokine interleukin-6. Biochim Biophys Acta.

[28] Lo C-W, Chen M-W, Hsiao M, Wang S, Chen C-A, Hsiao S-M (2011). IL-6 trans-signaling in formation and progression of malignant ascites in ovarian Cancer. Cancer Res.

[29] Lee SO, Yang X, Duan S, Tsai Y, Strojny LR, Keng P (2016). IL-6 promotes growth and epithelial-mesenchymal transition of CD133+ cells of non-small cell lung cancer. Oncotarget..

[30] Ando K, Takahashi F, Motojima S, Nakashima K, Kaneko N, Hoshi K (2013). Possible role for tocilizumab, an anti–Interleukin-6 receptor antibody, in treating Cancer Cachexia. J Clin Oncol.

[31] Maude SL, Frey N, Shaw PA, Aplenc R, Barrett DM, Bunin NJ (2014). Chimeric antigen receptor T cells for sustained remissions in leukemia. N Engl J Med.

[32] Jin H-T, Ahmed R, Okazaki T, Ahmed R, Honjo T (2011). Role of PD-1 in regulating T-cell immunity. Negative co-receptors and ligands.

[33] Ruf P, Kluge M, Jager M, Burges A, Volovat C, Heiss MM (2010). Pharmacokinetics, immunogenicity and bioactivity of the therapeutic antibody catumaxomab intraperitoneally administered to cancer patients. Br J Clin Pharmacol.

